# Micromorphological and phytochemical survey of *Ballota acetabulosa* (L.) Benth

**DOI:** 10.1111/plb.13254

**Published:** 2021-05-14

**Authors:** C. Giuliani, M. Bottoni, R. Ascrizzi, F. Milani, S. Falsini, A. Papini, G. Flamini, G. Fico

**Affiliations:** ^1^ Department of Pharmaceutical Sciences University of Milan Via Mangiagalli 25 I‐20133 Milan Italy; ^2^ Department of Pharmaceutical Sciences Ghirardi Botanic Garden University of Milan Via Religione 25 I‐25088 Toscolano Maderno, Brescia Italy; ^3^ Department of Pharmacy University of Pisa Via Bonanno 6 I‐56126 Pisa Italy; ^4^ Department of Biology University of Florence Via La Pira 4 I‐50121 Florence Italy

**Keywords:** Greek horehound, Lamiaceae, microscopy, HS‐SPME, trichome morphotypes, VOC profile

## Abstract

- Within the Open Science project entitled ‘Botanic Garden, factory of molecules’, a multidisciplinary study approach was applied to *Ballota acetabulosa* (L.) Benth., at the Ghirardi Botanic Garden (Toscolano Maderno, BS, Italy).
- Micromorphological and histochemical investigations were performed on the secreting structures of the vegetative and reproductive organs under light, fuorescence and electronic microscopy. Concurrently the characterization of the volatiles spontaneously emitted from leaves and flowers were examined.
- Four trichome morphotypes were identified: peltate and short‐stalked, medium‐stalked and long‐stalked capitate trichomes, each with a specific distribution pattern. The histochemical analysis was confirmed using ultrastructural observations, with the peltates and long‐stalked capitates as the main sites responsible for terpene production. The head‐space characterization revealed that sesquiterpene hydrocarbons dominated both in leaves and flowers, with γ‐muurolene, β‐caryophyllene and (*E*)‐nerolidol as the most abundant compounds. Moreover, a comparison with literature data concerning the ecological roles of the main compounds suggested their dominant roles in defence, both at the leaf and flower level.
- Hence, we correlated the trichome morphotypes with the production of secondary metabolites in an attempt to link these data to their potential ecological roles. Finally, we made the obtained scientific knowledge available to visitors of the Botanic Garden through the realization of new labelling dedicated to *B. acetabulosa* that highlights the ‘invisible’, microscopic features of the plant.

## Graphical abstract

In *Ballota acetabuolosa*, peltates and long‐stalked capitates are responsible for terpene production. γ‐Muurolene, β‐caryophyllene and (*E*)‐nerolidol prevail in the foliar and floral VOC profiles.

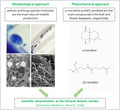

## Introduction

The genus *Ballota* L. (Lamiaceae) includes about 35 perennial herbs and subshrubs, native to the temperate regions of Asia, Africa and Europe, with its largest centre of diversity located in the Mediterranean Basin (Morteza‐Semnani & Ghanbarimasir 2019). Traditional medicine ascribes to them antiemetic, antispasmodic, sedative, vermifuge and antitussive properties (Yazgan *et al.* 2010; Morteza‐Semnani & Ghanbarimasir 2019).

*Ballota acetabulosa* (L.) Benth., known as Greek horehound, is a perennial herb native to Greece and the Aegean region, reaching up to 60–80 cm in height, woody at the base, with greyish tomentose stems covered with simple and stellate hairs; middle and upper cauline leaves, crenate‐dentate, with a petiole 5–15‐mm long; bracteoles linear to spathulate, membranous; the inflorescences are verticillasters, six to 12 flowered; the calyx is 12–15‐mm long and the corolla is 15–18‐mm long, purple and white in colour (Tutin *et al.* 1972).

In traditional Greek and Roman medicines, *B. acetabulosa* was an ingredient in the *Dictamnus*, the common name for a complex of medicinal species belonging to the Rutaceae and Lamiaceae families, used in the treatment of gynaecological disorders and affections of different origins (Martínez‐Francés *et al.* 2015).

Regarding the morphological and phytochemical characterization of the species, the available contributions are few. The micromorphological literature contains two dated investigations focused on the leaves (Psaras 1986; Psaras & Rhizopoulou 1995), and one study concerning the anatomy of the aerial vegetative organs, in which references to glandular and non‐glandular trichomes are reported (Yazgan *et al.* 2010).

In terms of phytochemical data, there are no studies focused on the profile of the volatile organic compounds (VOC), while several works on the essential oil composition of congeneric species exist, often linked to evaluations of their biological activity (Morteza‐Semnani & Ghanbarimasir 2019; Rosselli *et al.* 2019). Among the most recent works are investigations on *B. bullata*, *B. nigra* subsp. *uncinata* and subsp. *foetida*, *B. undulata*, *B. saxatilis* and *B. hispanica*, some of which refer to Italian samples (Riccobono *et al.* 2016; Rigano *et al.* 2017, 2020; El Mokni *et al.* 2020). With regard to *B. acetabulosa*, the literature offers several studies on the characterization of extracts of increasing polarity in relation to their potential biological activity; moreover, there are studies in which the composition of polyphenolic, flavonoidic and diterpenic content are analysed (Sahpaz *et al.* 2002; Couladis *et al.* 2003; Yilmaz & Çitoǧlu 2003; Dumlu & Bulut 2007; Dulger & Kilcik 2011a, 2011b; Dulger & Dulger 2012; Askun *et al.* 2013; Rosselli *et al.* 2019).

In this context, the present study aims to increase knowledge on *B. acetabulosa* in the micromorphological and phytochemical fields through complementary investigative approaches. The primary goals are: (i) description of the morphology and distribution pattern of non‐glandular and glandular trichomes by means of light (LM) and scanning electron microscopy (SEM); (ii) analysis, reported here for the first time, of the main compound classes secreted by glandular trichomes through histochemical assays; (iii) study of ultrastructural features of the secreting cells by means of transmission electron microscopy (TEM), in order to identify cellular compartments involved in the secretory process; (iv) characterization, as a novel element, of the VOC spontaneously emitted by leaves and flowers; and (v) correlation of the VOC profiles with potential ecological roles through analysis of literature data concerning the ecological action of the dominant compounds.

Finally, the present work is part of a wider project entitled ‘Botanic Garden, factory of molecules’, investigating a selection of species conserved at the Ghirardi Botanic Garden (Toscolano Maderno, BS, Italy) through a multi‐level study using micromorphological and phytochemical approaches in relation to the ecology and biological activity of the secondary metabolites. The achieved results have converged in the realization of new pictorial interpretations dedicated to *B. acetabulosa*, where results of the scientific research are made available to the general public.

## Material and methods

### Plant material

*Ballota acetabulosa* is conserved at the Ghirardi Botanic Garden (Toscolano Maderno, BS, Lombardy, Italy), Department of Pharmaceutical Sciences, University of Milan. Samplings for the micromorphological and phytochemical investigations were performed in June 2019. Voucher specimens were labelled with the code GBG2019/027 and deposited in the Herbarium of the Ghirardi Botanic Garden.

### Trichome investigations

The micromorphology, localization and histochemistry, together with ultrastructural features of trichomes on both vegetative and reproductive organs were studied by means of LM, fluorescence microscopy (FM), SEM and TEM.

We examined at least ten replicates, collected from the same individual, per each plant part to evaluate variability in the micromorphological features. Referring to trichome localization on the examined plant parts, we qualitatively evaluated their distribution using the following symbols: (−) absent, not observed in any of the replicates; (±) sporadic in no more than four replicates; (+) present in all replicates; (++) abundant in all replicates, with distribution on the whole organ surface.

#### Light microscopy (LM)

Both fresh and fixed samples were analysed. The fresh material was hand‐cut into sections 50–60‐μm thick; the fixed samples were soaked in FAA solution (formaldehyde/acetic acid/ethanol 70% = 5:5:90) for 7 days, then dehydrated in an ascending ethanol series up to absolute, embedded in Technovit/Historesin and sectioned with a microtome into sections 10–20‐μm thick. The following chemicals were used for histochemical staining: Toluidine blue as a general dye (Beccari & Mazzi 1966), Nadi reagent for terpenes (David & Carde. 1964), Ruthenium red for acid polysaccharides (Jensen 1962), Alcian blue for mucopolysaccharides (Beccari & Mazzi 1966) and ferric trichloride for polyphenols (Gahan 1984). Control procedures were concurrently performed.

#### Fluorescence microscopy (FM)

The following histochemical stains were employed on hand‐cut fresh samples: Fluoral yellow‐088 for total lipids (Brundrett *et al.* 1991) and Nile red for neutral lipids (Greenspan *et al.* 1985). Observations were made with a Leitz DM‐RB Fluo optical microscope equipped with a Nikon digital camera.

#### Scanning electron microscopy (SEM)

Small segments of leaves, sepals, petals and floral pedicels were fixed in FAA solution, as described above, for 10 days, dehydrated in an ascending ethanol series up to absolute and critical‐point dried. The samples were mounted on aluminium stubs, gold‐coated and observed under a Philips XL 20 SEM operating at 10 kV.

#### Transmission electron microscopy (TEM)

Small pieces of plant material were fixed in 2.5% glutaraldehyde in 0.1 M phosphate buffer, pH 6.8, and post‐fixed in 2% OsO_4_ in the same phosphate buffer, dehydrated and embedded in Spurr’s resin. Ultrathin sections were stained with uranyl acetate and lead citrate. The samples were observed under a Philips EM‐300 TEM.

## Phytochemistry

### Volatile organic compounds (VOCs)

Three fresh leaves and three fresh flowers per plant were cut and immediately inserted into separate glass vials of suitable volume for analysis of VOC.

### Headspace‐SPME sample analysis

The headspace sampling conditions were as reported in Ascrizzi *et al.* (2017). For the headspace samples, Supelco SPME (Solid Phase Micro‐Extraction) devices, coated with polydimethylsiloxane (PDMS, 100 μm) were used. The same new fibre, preconditioned according to the manufacturer’s instructions, was employed for all analyses. To ensure a stable temperature, sampling was conducted in an air‐conditioned room at 22 ± 1 °C; this temperature was chosen to avoid thermal damage to the plant material and, thus, release of any artificially induced volatiles. After 30 min of equilibration, the fibre was exposed to sample the headspace for 30 min. Both the equilibration and sampling times were experimentally determined to obtain optimal adsorption of the volatiles, and to avoid both under‐ and over‐saturation of the fibre and of the mass spectrometer (MS) ion trap. Once sampling was complete, the fibre was withdrawn into the needle and transferred to the injection port of the GC‐MS system. Both the sampling and desorption conditions were identical for all samples. Furthermore, control blanks were performed before each first SPME extraction and randomly repeated during each series. Quantitative comparisons of relative peak areas were performed between the same compounds in the different samples.

### Gas chromatography–electron impact mass spectrometry analysis (GC–EI‐MS)

The GC–EI‐MS analyses were performed with a Varian CP‐3800 gas chromatograph (Varian, Walnut Creek, CA, USA) equipped with an Agilent DB‐5 (Agilent Technologies, Santa Clara, CA, USA) capillary column (30 m × 0.25 mm; film thickness 0.25 μm) and a Varian Saturn 2000 ion trap mass detector (Varian). Analytical conditions were as follows: injector and transfer line temperatures, 220 ºC and 240 °C, respectively; oven temperature programmed to rise from 60 ºC to 240 °C, at 3 °C min^−1^; carrier gas, helium at 1 ml min^‐1^; splitless injection. The identification of constituents was based on a comparison of their retention times with those of authentic samples (when available), comparing their linear retention indices relative to a series of pure *n‐*hydrocarbons (C5–C25). Computer matching was also used against commercial (NIST 14 and ADAMS) and laboratory‐developed library mass spectra built up from pure substances and components of commercial essential oils of known composition as well as MS literature data (NIST, 2014).

## Results

### Trichome morphotypes and distribution patterns

**Fig. 1:**
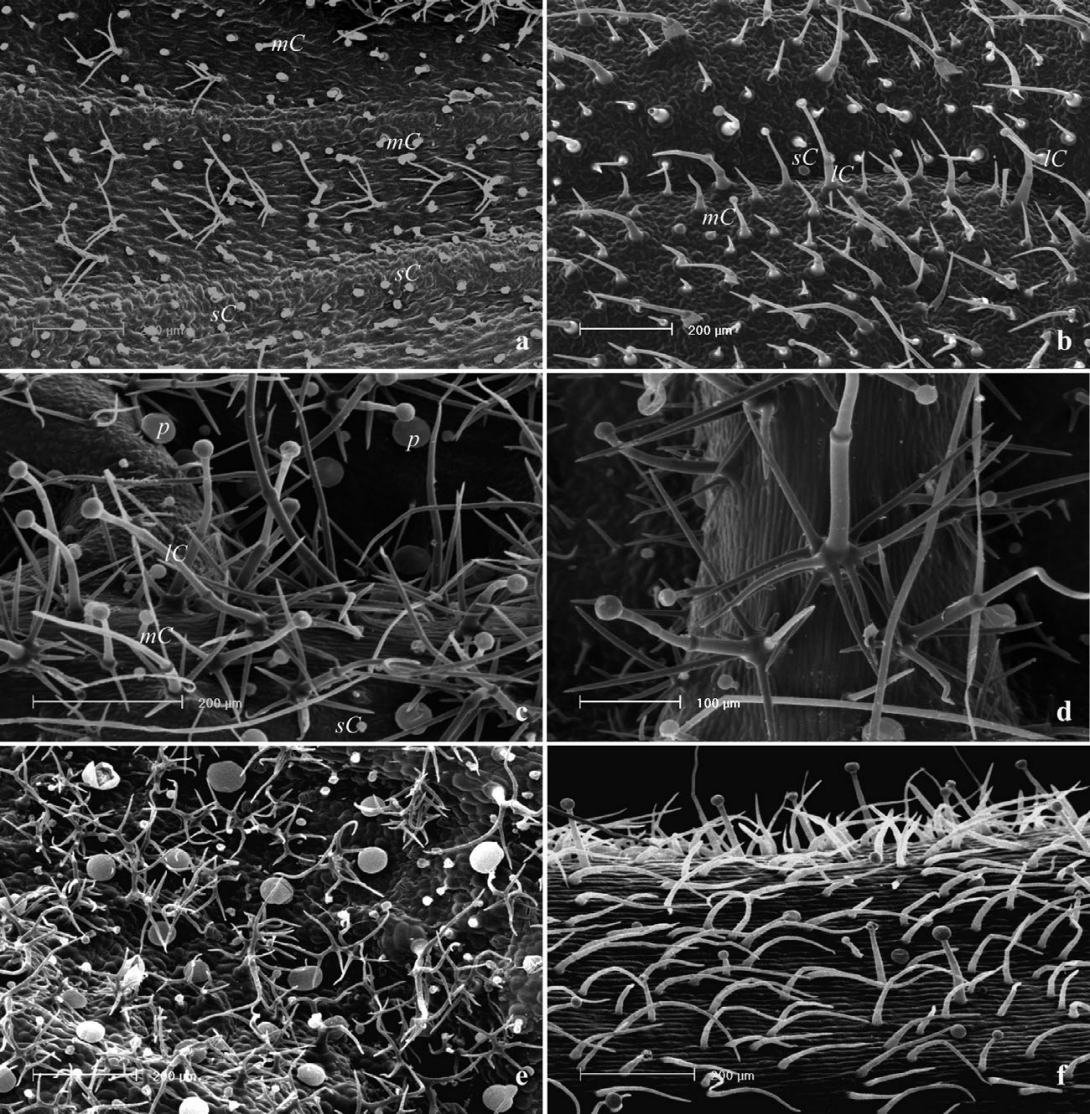
SEM micrographs showing trichome morphotypes and distribution patterns in *Ballota acetabulosa* (L.) Benth. (a) Leaf abaxial surface with abundant short capitates, occasional medium capitates and dendritic non‐glandular trichomes. (b) Leaf adaxial surface with short capitates, occasional medium and long capitates and simple non‐glandular trichomes. (c, d) Calyx abaxial surface: general view (c) and detail (d) with abundant peltates and the three morphotypes of capitates; notice the abundance of the dendritic trichomes. (e) Corolla abaxial surface at the apical region with peltates, short capitates and dendritic non‐glandular trichomes. (f) Floral pedicel with short capitates, long capitates and simple non‐glandular trichomes. *Symbols*: P, peltate; sC, short capitate; mC, medium capitate; lC, long capitate.

Leaves and flowers of *B. acetabulosa* displayed an indumentum of non‐glandular and glandular trichomes, the latter including both peltate and capitate types (Table 1, Fig. 1a–f). The peltate trichomes were composed of a basal cell, a stalk cell and a large secretory head with eight cells arranged in a single disc. They were localized throughout the whole plant epidermis, being scarce on leaves and common on the sepal and petal abaxial surfaces (Table 1, Fig 1c–e).

**Table 1:** Distribution pattern of the glandular and non‐glandular trichomes on abaxial (abax) and adaxial (adax) surfaces of *Ballota acetabulosa* (L.) Benth.

Trichome type	Leaf		Calyx		Corolla		Floral peduncle
	adax	abax	adax	abax	adax	abax	
peltate	±	±	−	++	−	++	±
short capitate	+	++	+	+	+	++	+
medium capitate	+	±	−	±	−	−	±
long capitate	±	−	−	+	−	−	+
simple non‐glandular	++	−	−	±	+	+	+
dendritic non‐glandular	−	+	−	+	−	+	−

Symbols: (−) missing, (±) sporadic, (+) present, (++) abundant.

Three different types of capitate trichome were found: short‐stalked consisting of a basal cell, a stalk cell and a round two‐ to four‐celled head, mainly located along the vein system of the whole plant, especially on the leaf and corolla abaxial sides (Table  1, Fig.  1a–c, e); medium‐stalked consisting of a basal cell, one to two stalk cells, a neck cell and a one‐ to two‐celled head, occurring on leaves and the calyx abaxial side (Table  1, Fig.  1a–c); and long‐stalked, consisting of a basal cell, two to four stalk cells, a neck cell and a globose two‐ to four‐celled secretory head. These hairs were primarily distributed on the leaf adaxial side, on the sepal abaxial side and on the floral peduncle (Table  1, Fig.  1b–d, f).

The observed non‐glandular hairs were of two different types. The first was simple, multicellular, uniseriate with distinct articulation between the cells (Fig.  1a–c, f), distributed on surfaces of both the vegetative and reproductive organs (Table  1). The second was dendritic multicellular hairs with four to six uniseriate arms (Fig.  1c–e), distributed on the calyx and the corolla abaxial side (Table  1).

### Trichome histochemistry and ultrastructure

**Fig. 2:**
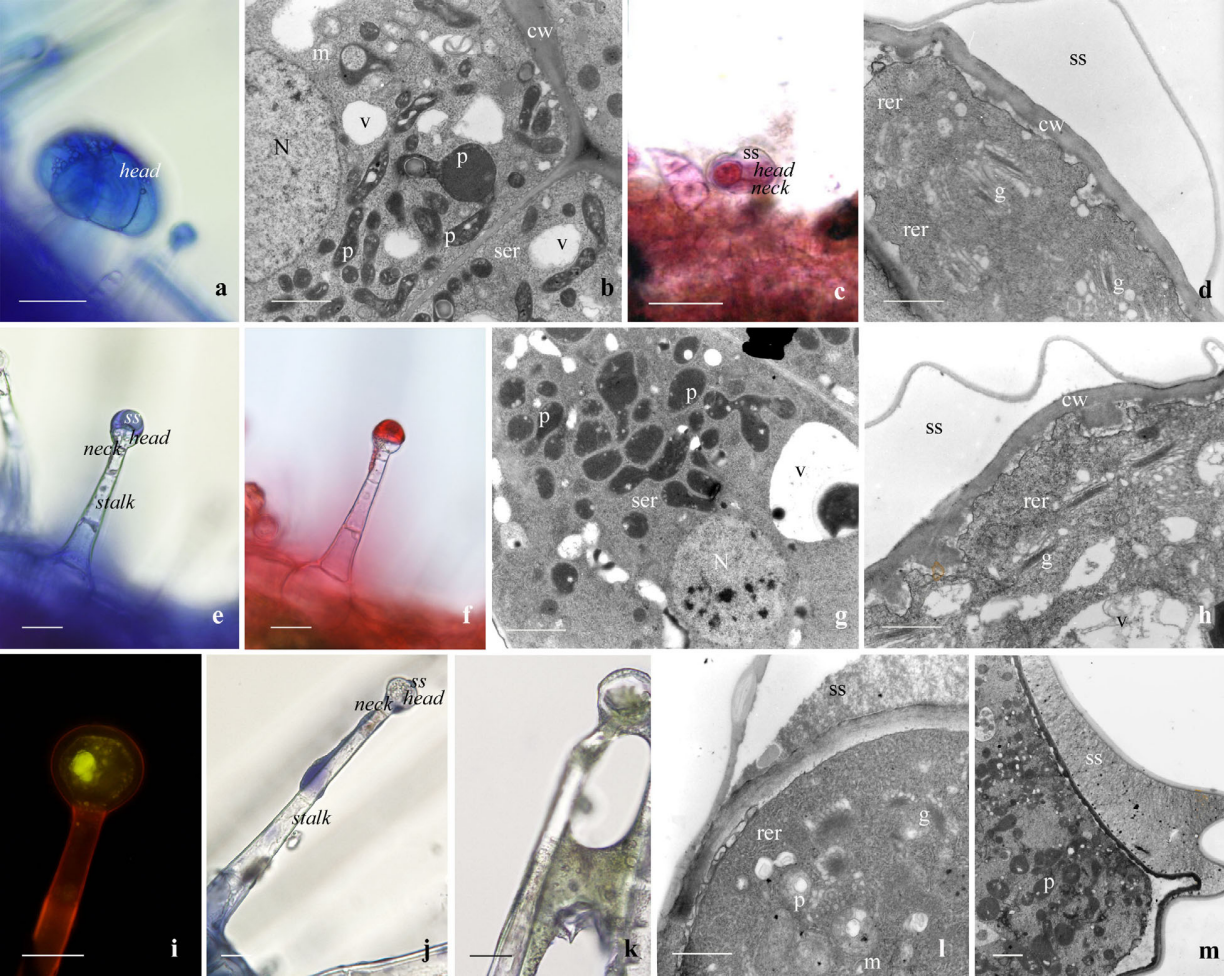
(a) Histochemistry of the peltate trichome of *Ballota acetabulosa*: Nadi reagent (LM). (b) Ultrastructure of the peltate trichome (TEM). (c) Histochemistry of the short‐stalked capitate trichome: Nadi reagent (LM). (d) Ultrastructure of the short‐stalked capitate trichome (TEM). (e, f) Histochemistry of the medium‐stalked capitate trichome: Nadi reagent (e), Ruthenium Red (f) (LM). (g, h) Ultrastructure of the medium‐stalked capitate trichome (TEM). (i‐k) Histochemistry of the long‐stalked capitate trichome: Nile Red (i), Nadi reagent (j), FeCl_3_ (k) (LM). (l, m) Ultrastructure of the long‐stalked capitate trichome at the beginning (l) and at the end (m) of the secretory process (TEM). *Scale bars*: a, c, e, f, i–k: 25 μm; b, d, g, h, l, m: 1 μm. *Abbreviations*: cw, cell wall; g, Golgi bodies; m, mitochondrion; N, nucleus; p, plastid; rer, RER; ss, subcuticular space; ser, SER; v, vacuole.

The results of the histochemical investigation on the glandular trichomes are reported in Table 2 and Fig. **2**.

**Table 2:** Results of the histochemical tests on the glandular trichomes of *Ballota acetabulosa* (L.) Benth. Peltates were scarce on leaves and present on sepal and petal abaxial surfaces; short capitates occurred throughout the plant, especially on the leaf and the corolla abaxial sides; medium capitates occurred on leaf and calyx abaxial sides; long capitates were present on leaf adaxial side and sepal abaxial side.

Stains	Target compounds	peltate	short capitate	medium capitate	long capitate
Fluoral yellow‐088	Total lipids	+	−	±	+
Nile red	Total lipids	+	−	±	+
Nadi reagent	Terpenoids	++	−	±	+
Ruthenium red	Acid polysaccharides	−	+	+	+
Alcian blue	Muco‐polysaccharides	−	±	+	+
Ferric trichloride	Polyphenols	−	−	−	+

Symbols: (−) negative response; (+) positive response; (±) weakly positive response; (++) intensely positive response.

In the peltate trichomes, all the employed lipophilic tests (Table 2, Fig. 2a) intensely stained the secreted material, whereas staining reagents such as Alcian blue and FeCl_3_ gave negative responses, indicating that the secretion is mainly composed of terpenes. In the secretory cell cytoplasm, the most frequent organelles were multi‐shaped, electron‐dense plastids and a well‐developed smooth endoplasmic reticulum (SER) (Fig. 2b).

In the short hairs, only the hydrophilic procedures gave faintly positive reactions, indicating the presence of polysaccharide components (Table 2, Fig. 2c); the FeCl_3_ test did not stain the secreted material. The secretory cell ultrastructure evidenced Golgi bodies and an abundant rough endoplasmic reticulum (RER) (Fig. 2d).

In the medium capitates, the secretion stained positively for both lipophilic and hydrophilic tests (Fig. 2e, f), whereas FeCl_3_ gave negative results. The trichome ultrastructure showed dense cytoplasm, numerous small vacuoles, electron‐dense plastids, few Golgi bodies and RER (Fig. 2 g, h).

In the long capitates, the simultaneous occurrence of terpenes, polysaccharides and phenolic compounds was evidenced, as indicated by positive responses to all the employed dyes (Table 2, Fig. 2i–k). The terpene and phenolic fractions were copiously produced and often observed flowing over the glandular head and along the stalk (Fig. 2j, k). At the beginning of the secretion phase, the cytoplasm of the glandular cells showed mitochondria, Golgi bodies, RER cisternae and plastids with starch granules; in fully active trichomes, Golgi bodies and RER elements became less common, plastids contained occasional small starch grains and appeared surrounded by dilated SER cisternae (Fig. 2l, m).

### Phytochemistry

#### Votatile organic compounds *(VOCs)*

The HS‐SPME analysis on leaves and flowers of *B. acetabulosa* revealed 46 compounds. In detail, 34 and 21 compounds were identified in the foliar and floral profiles, respectively (Table 3).

**Table 3:** HS‐SPME profiles of the leaves and flowers of *Ballota acetabulosa* (L.) Benth.

	l.r.i.^a^	Compounds	Relative Abundance (%)		Standard availability^b^	Chemical class^c^
			Leaves	Flowers		
1	1034	1,8‐cineole	tr	–	A	OM
2	1102	1‐nonanal	tr	5.28	A	FA
3	1143	geijerene	1.6	–	NA	SH
4	1199	*n*‐dodecane	tr	–	A	FA
5	1204	1‐decanal	0.12	7.51	A	FA
6	1241	methyl carvacrol	–	0.99	A	OM
7	1292	1‐tridecene	tr	–	A	FA
8	1340	δ‐elemene	0.19	–	NA	SH
9	1351	α‐cubebene	0.32	–	NA	SH
10	1368	cyclosativene	tr	–	NA	SH
11	1376	α‐copaene	2.22	2.65	A	SH
12	1380	daucene	0.69	–	NA	SH
13	1384	β‐bourbonene	3.54	tr	NA	SH
14	1390	β‐cubebene	0.96	1.59	NA	SH
15	1392	β‐elemene	1.96	2.63	A	SH
16	1399	*n*‐tetradecane	–	2.04	A	FA
17	1410	α‐gurjunene	0.26	–	A	SH
18	1415	1,7‐*di*‐*epi*‐β‐cedrene	0.18	–	NA	SH
19	1420	β‐caryophyllene	21.97	6.76	A	SH
20	1429	β‐copaene	2.16	–	M	SH
21	1433	γ‐elemene	–	3.78	NA	SH
22	1438	*trans*‐α‐bergamotene	0.85	–	M	SH
23	1439	α‐guaiene	0.82	–	NA	SH
24	1440	*cis*‐α‐ambrinol	–	1.29	NA	AC
25	1441	aromadendrene	–	3.12	A	SH
26	1453	(*E*)‐geranyl acetone	–	6.65	A	AC
27	1454	α‐*neo*‐clovene	0.12	–	NA	SH
28	1460	(*E*)‐β‐farnesene	5.12	tr	A	SH
29	1461	*allo*aromadendrene	2.53	–	M	SH
30	1462	*cis*‐muurola‐4(14),5‐diene	1.02	–	NA	SH
31	1477	γ‐muurolene	43.63	25.33	M	SH
32	1490	(*E*)‐β‐ionone	–	3.27	A	AC
33	1492	valencene	3.19	–	A	SH
34	1498	α‐muurolene	0.36	–	NA	SH
35	1500	*n*‐pentadecane	–	1.79	A	FA
36	1507	(*E,E*)‐α‐farnesene	1.35	–	M	SH
37	1513	*trans*‐γ‐cadinene	0.47	–	NA	SH
38	1524	δ‐cadinene	0.98	–	NA	SH
39	1529	lilial	–	0.88	A	FA
40	1538	α‐cadinene	0.12	–	NA	SH
41	1565	(*E*)‐nerolidol	–	17.14	A	OS
42	1575	germacrene D‐4‐ol	0.12	–	NA	OS
43	1581	caryophyllene oxide	0.21	–	A	OS
44	1594	carotol	2.04	–	M	OS
45	1600	*n*‐hexadecane	–	2.01	A	FA
46	1700	*n*‐heptadecane	–	2.34	A	FA
		Oxygenated monoterpenes	–	0.99		
		Sesquiterpene hydrocarbons	96.61	45.86		
		Oxygenated sesquiterpenes	2.37	17.14		
		Apocarotenoids	–	11.21		
		Fatty acid derivatives	0.12	21.85		
		Total	99.10%	97.05%		

^a^ Linear retention index (DB‐5 column).

^b^ A = available; NA = not available; M = available in mixture.

^c^ OM = Oxygenated monoterpene; SH = Sesquiterpene hydrocarbon; OS: Oxygenated sesquiterpene; AC = Apocarotenoid; FA Fatty acid derivative.

Almost all of the leaf profile was dominated by sesquiterpene hydrocarbons (96.61%), followed by oxygenated sesquiterpenes (2.37%) and non‐terpene derivatives (0.12%). The most abundant compound was γ‐muurolene (compound *31*, 43.63%), followed by β‐caryophyllene (*19*, 21.97%). (*E*)‐β‐Farnesene (*28*, 5.12%), β‐bourbonene (*13*, 3.54%), valencene (*33*, 3.19%), *allo*aromadendrene (*29*, 2.53%), α‐copaene (*11*, 2.22%), β‐copaene (*20*, 2.16%) and carotol (*44*, 2.04%) showed percentage relative abundances in the range 5.0–2.0%, while β‐elemene (*15*, 1.96%), geijerene (*3*, 1.60%), (*E,E*)‐α‐farnesene (*36*, 1.35%) and *cis*‐muurola‐4(14),5‐diene (*30*, 1.02%) in the range of 2.0–1.0%. The remaining compounds occurred at less than 1.0% or in trace amounts (<0.1%). A total of 25 exclusive compounds were identified, among which valencene (*33*, 3.19%), *allo*aromadendrene (*29*, 2.53%), β‐copaene (*20*, 2.16%) and carotol (*44*, 2.04%) were the most abundant. The remaining exclusive compounds accounted for less than 2.0% or trace amounts (<0.1%).

Sesquiterpene hydrocarbons represented the main compound class in the floral profile, (45.86%), followed by non‐terpene derivatives (21.85%), oxygenated sesquiterpenes (17.14%) and apocarotenoids (11.21%), while the monoterpene class contained only oxygenated compounds (0.99%). γ‐Muurolene (*31*, 25.33%) was the dominant compound, followed by (*E*)‐nerolidol (*41*, 17.14%), 1‐decanal (*5*, 7.51%), β‐caryophyllene (*19*, 6.76%), (*E*)‐geranyl acetone (*26*, 6.65%) and 1‐nonanal (*2*, 5.28%). γ‐Elemene (*21*, 3.78%), (*E*)‐β‐ionone (*32*, 3.27%), aromadendrene (*25*, 3.12%), α‐copaene (*11*, 2.65%), β‐elemene (*15*, 2.63%), *n*‐heptadecane (*46*, 2.34%), *n*‐tetradecane (*16*, 2.04%) and *n*‐hexadecane (*45*, 2.01%) showed abundances in the range 3.0–2.0%, while *n*‐pentadecane (*35*, 1.79%), β‐cubebene (*14*, 1.59%) and *cis*‐α‐ambrinol (*24*, 1.29%) were detected in relative abundances ranging between 2.0% and 1.0%. The other compounds were present at percentages below 1.0% or in trace amounts (<0.1%). Twelve exclusive compounds were detected, among which (*E*)‐nerolidol (*41*, 17.14%) and (*E*)‐geranyl acetone (*26*, 6.65%) were the most abundant. The remaining compounds were detected at percentages in the ranges 3.0‐2.0%, 2.0‐1.0% and < 1.0%.

Leaves and flowers shared nine common compounds: γ‐muurolene was most prevalent (*31*, 43.63% leaves; 25.33% flowers), followed by β‐caryophyllene (*19*, 21.97% leaves; 6.76% flowers), both with a higher percentage in leaves than in flowers. (*E*)‐β‐Farnesene (*28*) and β‐bourbonene (*13*) were more abundant in the leaves (5.12%; 3.54%), while in flowers they were only detected in trace amounts (<0.1%). In contrast, *n*‐decanal (*5*) and *n*‐nonanal (*2*) revealed higher percentages in flowers (7.51%; 5.28%) than in leaves (0.12%; traces, <0.1%), while the relative abundances of compounds (*11*), (*14*) and (*15*) were between 2.0–1.0% and they were quantitatively comparable in the two profiles.

### Scientific dissemination

**Fig. 3:**
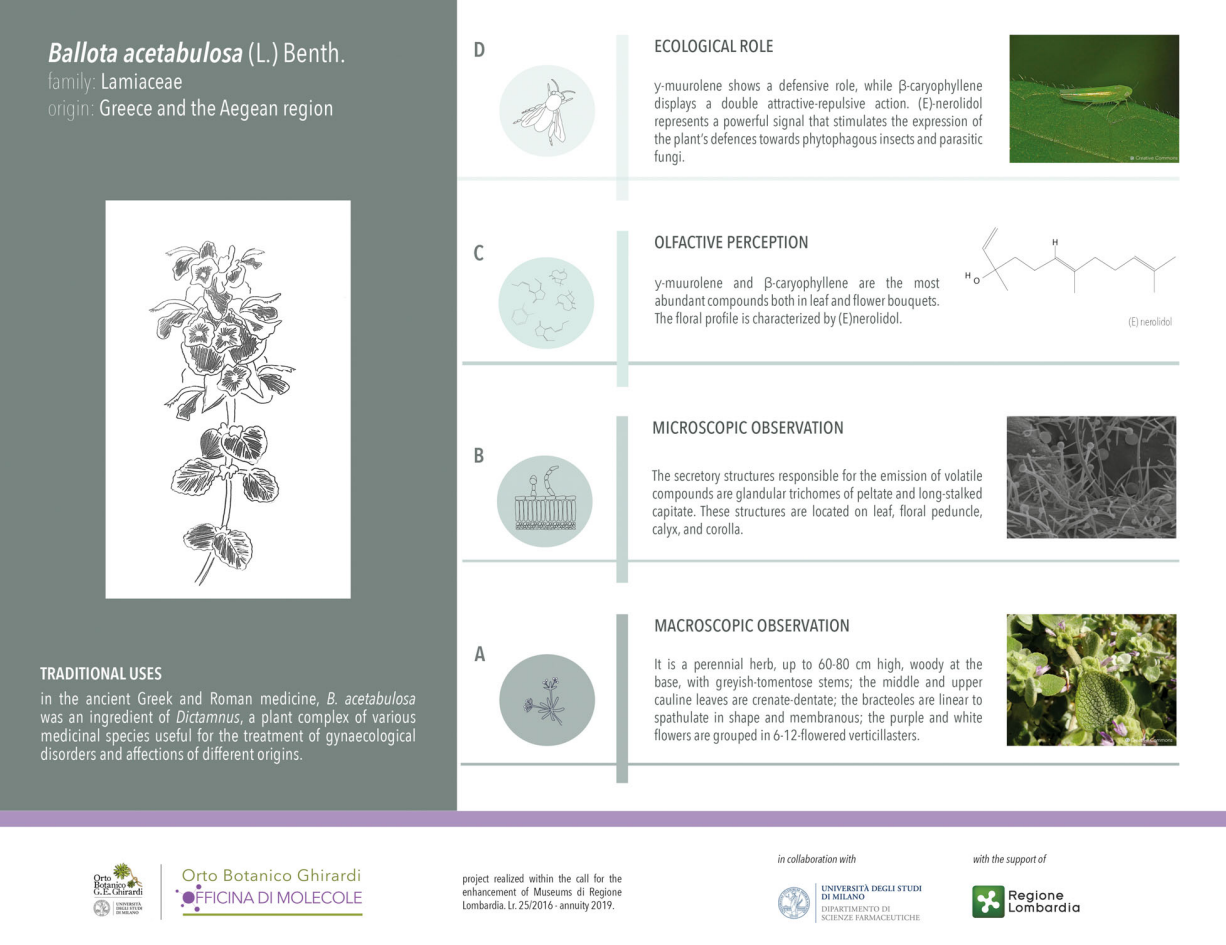
New labelling of *Ballota acetabulosa* (L.) Benth. at the Ghirardi Botanic Garden (University of Milan, Toscolano Maderno, Brescia, Italy).

The scientific results reported in the “Trichomes” and “Phytochemistry” sections converged in the realization of a new pictorial explanatory approach for *B. acetabulosa* at the Ghirardi Botanic Garden (Toscolano Maderno, BS, Italy). In addition to the plant macroscopic features, this highlights the microscopic morphology, volatile composition of the vegetative and floral bouquets, and noteworthy data on the plant–environment interactions (Fig. 3).

## Discussion

Based on their morphology, the glandular trichomes of *B. acetabulosa* corresponded to the two main types that occur in the Lamiaceae: peltate and capitate hairs.

The peltate hairs were widespread on the examined vegetative and reproductive organs, being scarce on leaves and abundant on sepals; they exhibited an eight‐celled head, as already described in *Scutellaria brevibracteata* subsp. *subvelutina* (Rech. f.) Greuter & Burdet (Giuliani *et al.* 2020, 2020a). The secreted substances were stained intensely only in response to the lipophilic dyes, especially the Nadi reagent, indicating that the secretion was exclusively composed of terpenes. Therefore, these hairs were typical essential oil producers, as found in many Lamiaceae (Werker 2000), although in several species of this family chemical complexity of the secretion has also been reported (Giuliani & Maleci Bini 2008). The ultrastructural study on secretory cells confirmed the histochemical evidence; indeed, the combination of SER and multi‐shaped electron‐dense plastids with few internal membrane systems is very common for terpenoid‐producing cells (Hallahan 2000). The combination of SER with electron‐dense plastids can be considered a sign of terpenoid production, even if the role of the SER is not yet completely understood (Levine 2004; Giuliani *et al.* 2017). The transfer of terpenoids from one organelle to another and, eventually, to the plasma membrane is mediated by membrane contact (Levine 2004). The membrane contacts between electron‐dense plastids and SER observed here may thus be considered an indication of the activity of terpenoid production.

*Ballota acetabulosa* presented three types of capitate hairs, which greatly differed in overall size, stalk length and shape of the secretory head. The short capitate trichomes were widespread and are the only type common to all Lamiaceae species examined so far (Giuliani & Maleci Bini 2008). The hydrophilic procedures only gave faint positive reactions, indicating the exclusive presence of muco‐polysaccharides in the secretion. During the secretory process, the ultrastructure of the head cells evidenced abundant Golgi bodies and proliferation of RER elements. The development of such compartments can be correlated with the synthesis of polysaccharides (Giuliani & Maleci Bini 2008). In most Lamiaceae species examined so far, these trichomes are rich in polysaccharides; nevertheless, in several members of *Stachys* subgenus *Betonica* (L.) Bhattacharjee and in *Stachys annua* (L.) L. the secretion is more complex in composition, containing both terpene and polyphenolic fractions (Giuliani & Maleci Bini 2008).

The medium capitate trichomes corresponded to the capitate type‐II hairs described in several members of the genera *Salvia, Stachys, Sideritis* and *Scutellaria* (Giuliani & Maleci Bini 2008; Giuliani *et al.* 2020a, 2020b). The resulting secretion was heterogeneous, since it stained positively in both lipophilic and hydrophilic tests. The trichome ultrastructure displayed dense cytoplasm with numerous small vacuoles, electron‐dense plastids, few Golgi bodies and RER, confirming the histochemical results.

The long capitate hairs were scarce on leaves and numerous on the calyx abaxial surface. Noteworthy, these capitate morphotypes were distributed on leaves in our samples, while in the other Lamiaceae species examined so far they were found only on reproductive organs (Giuliani & Maleci Bini 2008). The secreted material stained positively for all the employed staining procedures, revealing the production and release of a heterogeneous secretion consisting of lipid, polysaccharide and phenolic compounds. Secretory cell ultrastructure supports the histochemical observations; indeed, at the beginning of the secretory phase, the head cells showed mitochondria, Golgi bodies and well‐developed RER elements, with plastids containing some starch grains. Plastids are also involved in the production of polyphenol precursors, and the key enzymes for their biosynthesis are localized in the starch granules (Grundhöfer *et al.* 2001). In mature trichomes, Golgi bodies and RER occurred only occasionally, while the most striking ultrastructural features were multi‐shaped plastids sheathed by periplastidial SER. These ultrastructural features may indicate the initial production of hydrophilic substances and polyphenols, followed by the release of terpenes at a later stage of the secretory process (Giuliani & Maleci Bini 2008).

The observed glandular trichome morphotypes were consistent with those previously reported for the target species from Turkey (Yazgan *et al.* 2010), although a comparison of the distribution pattern was not possible, and from several congeneric species from Egypt (Osman 2012). Regarding the non‐glandular indumentum, our observations confirmed the occurrence of simple uniseriate trichomes on the whole plant epidermis and of dendritic trichomes exclusively on the reproductive organs. However, we did not detect the stellate morphotype, previously reported in *B. acetabulosa* (Tutin *et al.* 1972; Yazgan *et al.* 2010) and in other congeneric species (Osman 2012). This could be significant and was probably related to the cultivation conditions in the study area, which could have affected the features of the non‐glandular indumentum.

Concerning the phytochemical investigations, the foliar profile was more complex than the floral profile, as also evidenced by the presence of a higher number of exclusive compounds, compared to that of the flowers (25 *versus* 12). Moreover, only nine common compounds were detected, among which the most abundant were: γ‐muurolene, with a relative percentage higher in leaves than in flowers (*31*, 43.63% leaves; 25.33% flowers), and β‐caryophyllene, the second most abundant compound in leaves (*19*, 21.97%), while in flowers it was less abundant (*19*, 6.76%). As for the exclusive compounds, valencene (*33*, 3.19%) was the most abundant in leaves, while in flowers (*E*)‐nerolidol (*41*, 17.14%) was most prevalent.

In general, in the case of Lamiaceae species, flowers emit more compounds than leaves. However, the opposite situation is not uncommon. For example, the leaf profile was more complex than the floral profile in *Scutellaria altissima* (Giuliani *et al.,* 2020, 2020b), *Salvia mirzayanii* (Majid, 2015) and *Lamium hybridum* (Flamini *et al.,* 2005).

The comparison between phytochemical and histochemical results confirms the terpene production, in particular on the petal abaxial surface, where peltate trichomes occurred, and on the calyx and floral peduncle, where long‐stalked capitates were detected. In contrast, there was no such marked correspondence for the leaves, where terpene productivity appeared to be related to the presence of medium‐stalked capitates. Due to the absence of studies on the VOC and essential oil (EO) characteristics of the target species, only a comparison with some congeneric species is possible. Specifically, studies carried out in Italy investigated the EO composition of *B. hispanica*, *B. nigra* subsp. *uncinata* and subsp. *foetida*, *B. undulata* and *B. saxatilis* (Fraternale *et al.* 2009; Fraternale & Ricci 2014; Riccobono *et al.* 2016; Rigano *et al.* 2017, 2020). Despite the detailed technical approaches used in this study, the volatile profile provided suggests the need for further studies on *B. acetabulosa* volatile profile using *in vivo* techniques.

Concerning the ecological role(s) of the main compounds detected in the VOC profiles, it emerged that the most abundant compound, γ‐muurolene (*31*), has a role in defence, and is the most common sesquiterpene hydrocarbon (Chizzola 2013; Giuliani *et al* *.,* 2020, 2020b). Although it was detected as a common compound, its high relative abundance in the leaves allowed us to hypothesize a marked repellent action at the vegetative organ level. Regarding another common compound, β‐caryophyllene (*19*), is known to act in both attraction and repulsion (Abraham *et al.* 2018; Zhang 2018; Lobo *et al.* 2019; Giuliani *et al.* 2020a, 2020b). Indeed, it attracts honeybees such as *Apis cerana* (Abraham *et al.* 2018), non‐pollinators such as *Vespa velutina* and species in the genus *Bombus* (Zhang 2018), but it provides defence against parasites such as *Diaphania hyalinata* (Lobo *et al.* 2019), hemiptera and lepidoptera in the genera *Apolygus*, *Aphis* and *Helicoverpa* (Zhang *et al.* 2020). Therefore, in our case study, we hypothesize that this compound has an attraction role in flowers and a repellent role in leaves. The plants must face two simultaneous and conflicting issues: the need to advertise their pollen as a reward for pollinating insects and the necessity to defend it from predators.

Concerning the leaf exclusive compounds, *allo*aromadendrene (*29*), together with compounds including β‐copaene (*20*), have larvicidal activity towards *Culex quinquefasciatus* (Senthilkumar *et al.* 2008). In addition, in an ecological survey conducted at the community level, it was documented that the abundance of this compound increases in cases of interspecific competition (Ormeño *et al.* 2007).

Referring to the floral bouquet, the overall abundance of sesquiterpene hydrocarbons makes it difficult to exclude a defence action even at the flower level. To this end, the exclusive compound (*E*)‐nerolidol (*41*) represents a powerful signal that stimulates expression of plant defences. In fact, its production activates a consequential series of cellular and molecular reactions, leading the plant to synthetize and accumulate a significant amount of broad‐spectrum defence molecules against herbivores and several types of parasite (Chen *et al.* 2020). Nerolidol is synthesized in many plants as an intermediate in the production of a herbivore‐induced terpenoid, (*E*)‐4,8–dimethyl–nonatriene (DMNT) (Chan *et al.* 2016). Moreover, it could even develop an antibacterial action together with β‐farnesene (*28*) (Kiryu *et al.* 2018), and also act as a nematicide (El‐Habashy *et al.* 2020).

In addition, *(E)*‐geranyl acetone (*26*) is involved in several VOC‐mediated tritrophic interactions (Morawo *et al.* 2016; Pinto‐Zevallos *et al.* 2018; Giuliani *et al.* 2020a, 2020b). This suggests the need to accumulate potential defence compounds against target species of flower parasites. Overall, the data stimulated our curiosity for future investigations on possible parasites known from the literature in relation to *B. acetabulosa*, as information is lacking on such aspects.

Finally, our study, which originated and takes place at the Ghirardi Botanic Garden, converged towards the fruition of research products available to the general public, following a new approach for dissemination of scientific data that is increasingly available in the literature (Giuliani *et al.* 2020, 2020b, 2020a, 2020b).

## Conclusion

Our work on *B. acetabulosa* was based on a multidisciplinary approach. In the micromorphological survey, the occurrence of peltate trichomes and of three capitate morphotypes emerged, each characterized by a specific distribution pattern on the vegetative and reproductive organs. The application of histochemical tests was an element of novelty for the target species; the results were confirmed by the ultrastructural observations, corroborating that the peltates and long‐stalked capitates are mainly responsible for terpenes production, finally resulting in the spontaneous emission of volatiles, as characterized through head‐space analysis in the phytochemical survey. In the vegetative and floral bouquets, the dominance of sesquiterpene hydrocarbons emerged, among which γ‐muurolene, β‐caryophyllene and (*E*)‐nerolidol were the dominant compounds. Moreover, a comparison with literature data on the ecological roles of the most abundant compounds allowed us to hypothesize a prevailing defence action of the volatile emissions, both at the leaf and flower level. These data represent a new focus in the research pathway towards characterization of the Ghirardi Botanic Garden as a ‘factory of molecules’, an innovative vision placed in an Open Science context.

## Funding

The authors are grateful to the Lombardy Region for the financial support of the project “Botanic Garden, factory of molecules”, under the Call for the Enhancement of Museums Lr. 25/2016, year 2019.
